# For Special Issue “Molecular Mechanisms of Responses to Low-Intensity Exposures 2.0” of *International Journal of Molecular Sciences*

**DOI:** 10.3390/ijms24087665

**Published:** 2023-04-21

**Authors:** Nadezhda S. Kudryasheva

**Affiliations:** 1 Institute of Biophysics SB RAS, Federal Research Center “Krasnoyarsk Science Center” SB RAS, Krasnoyarsk 660036, Russia; kudr@ibp.ru; 2 Biophysics Department, Siberian Federal University, Krasnoyarsk 660041, Russia

The intention of this Special Issue is to highlight the peculiarities of low-intensity/low-concentration exposures for organisms and to examine the molecular mechanisms of the organismal responses.

Low-intensity exposures are the most unexplored field of modern molecular toxicology. A lack of knowledge on the mechanisms of low-intensive factors causes problems in (1) the prediction of biological effects, (2) overcoming negative consequences, and (3) the application of positive results. Therefore, the analysis of low impacts is topical from both fundamental and applied standpoints, particularly important for ecology, biology, and medicine.

Studies of the biological effects of low-dose exposures have been conducted since the 1960s [1]. The works of Calabrese (Laboratory of Toxicology, University of Massachusetts Settlement, USA) are widely known [2,3]. Modern toxicology uses three dose–response models: linear, threshold, and hormesis. The latter implies an activation of physiological functions at low-dose exposures and their inhibition at higher doses; the model describes these effects in terms of ‘adaptive response’ and ‘toxicity’, respectively. It is supposed that the hormesis model can be applied as a basic one, transforming to the other models under definite restrictions.

Studies of biological responses to various bioactive compounds and the radiation of different types under the conditions of low-intensity exposures were encouraged in this Special Issue. A chemical and biochemical basis for these responses was of interest. The results presented contribute to understanding the molecular mechanism of “hormetic” responses to low concentrations of bioactive compounds and low-intensity radioactivity. One of the findings of this Special Issue is that the time of low-intensity exposure is a critical parameter in hormetic responses, along with the dose and type of active compounds [4]. Similar conclusions were reported previously by Sthijns and coauthors [5].

The biomedical aspect of low-intensity exposure is the most evident in the papers published in the Special Issue. For example, antimicrobial, anti-inflammatory, and tissue-stimulating effects of cold argon atmospheric plasma (CAAP) accelerate its use in various fields of medicine. Ermakov and coworkers [6] investigated the effects of CAAP of different radiation doses on mesenchymal stem cells and human osteosarcoma cells. The effects of low and high doses of CAAP treatment on normal and cancer cells were considered in terms of hormesis phenomenon. The authors found that the low dose of cold argon plasma irradiation stimulated the vital processes in stem cells, and they attributed this effect to the slight generation of reactive oxygen species. Oppositely, in cancer cells, the same doses lead to the formation of oxidative stress, which was accompanied by cell death. It is hoped that such a selective effect of cold argon atmospheric plasma can be used in the combined therapy of oncological diseases.

The work of Hanson and coworkers [7] focuses on hyper-radiosensitivity in cell lines. It was stated previously that the hyper-radiosensitivity of cells is decreased due to preliminary low-dose exposures; the mechanism of this phenomenon was supposed to be dependent on transforming growth factor β3. The current results contribute to the understanding of the mechanism of the transforming-growth-factor-β3-mediated removal of hyper-radiosensitivity.

Kovel [8] and Sushko [9] studied the bioeffects of perspective antioxidants and catalyzers, fullerenols, and water-soluble polyhydroxylated derivatives of fullerene. The toxicity and antioxidant activity of the fullerenols were under consideration. The authors demonstrated the advantages of a bacteria-bioluminescence-based bioassay to monitor and compare the properties of fullerenols; Gd-containing fullerene derivative and “emply” fullereno were applied as examples. Hormetic effects were found when bacterial cells were exposed to low-concentration solutions of fullerenols, involving conditions of model oxidative stress. The bioluminescence activation stage (stage II) in the presence of Gd-containing fullerenol is evident in Figure 1.

The advantages of bacterial bioluminescent bioassays were applied while studying the bioactivity of iron oxide nanoparticles [11] and different types of low-dose radioactivity [4].

Under the conditions of model oxidative stress, the bacterial bioassay revealed prooxidant activity, with a corresponding decay in the content of reactive oxygen species [11]. The results also indicated that cell membrane processes are responsible for the bioeffects and bacterial generation of reactive oxygen species.

The review by Kolesnik and coauthors [4] focused on low-dose bioeffects of a series of alpha and beta emitting radionuclides (americium-241, thorium-232, uranium-(235 + 238), and tritium), as well as gamma radiation. The applicability of hormetic and threshold models was discussed for radionuclides and gamma radiation, respectively. Dependences of the bacterial luminescence response on the irradiation intensity and exposure time were reviewed under the conditions of low-dose exposures. Several aspects of molecular intracellular mechanisms under low-intensity irradiation were analyzed: changes in the rates of enzyme processes in the bacterial cells, the consumption of intracellular reducers, the active role of reactive oxygen species, and DNA repairing. The radioprotector’s function of humic substances is also discussed in the review.

As an outline, the current Special Issue combined successfully research works which elucidated the molecular mechanisms of organismal low-dose/low-intensity responses to different physico-chemical factors, such as cold argon atmospheric plasma, low-dose radiation, and nanomaterials of different structures.

## Figures and Tables

**Figure 1:**
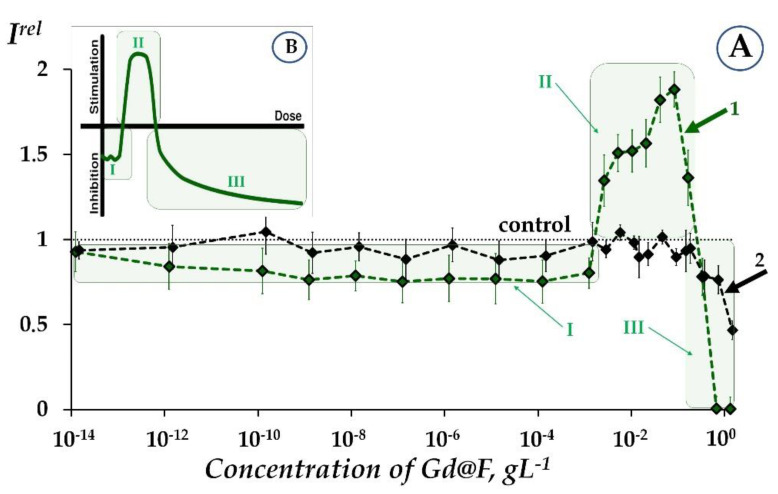
(**A**) Relative bioluminescence intensity, *I^rel^*, at different concentrations of Gd-containing fullerenol in bacterial suspension (1) and enzymatic system (2). (**B**) Scheme of hormesis dose–effect model is presented according to Rozhko et al. [10]. Hormetic stages: I—stress recognition, II—physiological activation, III—inhibition of vital functions. The figure was reproduced from Sushko et al. [9].
